# The process of spatial knowledge acquisition in a square and a circular virtual environment

**DOI:** 10.2478/v10053-008-0003-6

**Published:** 2008-07-15

**Authors:** Petra Jansen-Osmann, Martin Heil

**Affiliations:** 1 Department of Experimental Psychology, Heinrich-Heine-Universität Düsseldorf, Germany

**Keywords:** spatial cognition, spatial knowledge, children, development, virtual environments

## Abstract

This study investigated the effect of the environmental structure (circular vs. square environment) on spatial knowledge acquisition in a desktop virtual situation in which self-determined movement was allowed with a total of 120 participants: 7-, 8-year-old children; 11, 12-year-old children, and adults. In all measurements of spatial knowledge acquisition an overall developmental performance increase from younger children to adults was found. In contrast to that, the exploration and learning behavior did not differ between adults and children. Furthermore, the environmental structure influencedthenumber of trials needed to learn the two routes used and the distance walked to the determined landmarks. All these tasks were easier in a circular than in a square environment. This influenceofthe environmental structure was absent in the direction estimations task. The advantage of spatial knowledge acquisition in a circular environment in three of four tasks is discussed.

## INTRODUCTION

It is the main goal of this study to investigate the influence of the environmental structure on the process of spatial knowledge acquisition for adults as well as for children in a large-scale or environmental space, i.e., a space which is not perceivable from one single vantage point (see e.g., Canter & Craig, 1981). One factor often neglected when investigating the acquisition of survey knowledge concerns the so-called “carpentered world hypothesis” . It suggests that people living in highly industrialized environments perceive angles and straight edges differently from people who live in environments without square, manufactured structures (see e.g., Allport & Pettigrew, 1957). The different perception of angles and straight edges depending on the environment is evident in all processing stages during spatial knowledge acquisition, that is, from (1) exploring and (2) learning a route in an unknown environment up to (3) the acquisition of survey knowledge of the respective environment.

Additionally, the so-called “environmental legibility” (Lynch, 1960) may play a role for spatial knowledge acquisition. This concept describes the ease with which people can understand the layout of a place. Drawing upon extensive studies conducted in Boston, Jersey City, and Los Angeles, Lynch analyzed the legibility of the following easily recognizable five elements in the environmental space: paths, edges, districts, nodes, and landmarks. A more theoretical approach concerning the influence of the environmental structure on spatial knowledge acquisition is the regularity hypothesis by Thorndyke and Hayes-Roth (1982) . This hypothesis assumes that the regularity of an environment, that is, a route with straight paths and mostly right angles, affects how rapidly a person is able to learn the spatial relationships. If an environment is regular, locations might be determined by a coordinated frame of reference, whereby the entire environment is coded in relation to abstract axes defining the grid (Hart & Moore, 1973; Piaget & Inhelder, 1967). In an irregular environment, however, a coordinated frame of reference is difficult to use. Although the regularity hypothesis describes the structural influence in an environmental space on a theoretical level, the empirical evidence regarding this influence is scarce. Several studies investigated its impact on spatial knowledge with adults (Ruddle & Péruch, 2004; Werner & Schindler, 2004; Werner & Schmidt, 1999), but they all focused on separate aspects in the process of spatial cognition acquisition. The impact of the regularity hypothesis with children was only investigated by Herman, Blomquist, and Klein (1987) ; and by our own group (Jansen-Osmann, Schmid, & Heil, 2007a, 2007b). 

Herman et al. (1987) examined the spatial knowledge acquisition of 8-year-old children, 11-year-old children, and adults in environments with either a square or a curved structure. Both environments were symmetrical and only differed with respect to the kind of angles (almost right vs. beveled) and kind of paths (straight vs. curved). Participants were driven through the environments three times in an automobile and made direction and distance estimations to target locations after each trip. Eight-year-old children had more difficulties than older children and adults, but performance improved as subjects became increasingly familiar with the environment. Most importantly, however, the structure of the environment did not have an effect on participants’ performance. Several factors may account for this result. First of all, although the environments differed with respect to the kind of angles, both were symmetrical. Second, only some aspects of spatial knowledge (i.e., direction and distance estimations) were taken into account, while others like configurational measurements (drawing of a map), were completely ignored. Third, the ability to learn a route was not investigated at all. And finally, participants were not allowed to explore the environment on their own, which is critical due to the well known result that self-determined exploration facilitates spatial knowledge acquisition especially for younger children (Feldmann & Acredolo, 1979; Herman, Kolker, & Shaw, 1982). 

For these reasons we recently conducted two studies in which the effects of the symmetry of the environmental structure on the spatial acquisition process was investigated in more detail in a desktop virtual environment in which self-determined movement was allowed. In both studies symmetry was varied by using a square environment and another one where the routes were beveled and the right upper edge was missing. In our first study (Jansen-Osmann et al., 2007a) an overall developmental achievement from younger children to adults was found. Only the exploration behavior did not differ between adults and children. Furthermore, the environmental structure tended to influence only the learning behavior of younger children: They needed more learning trials in an asymmetrical than in a symmetrical environment. The environmental structure, however, did not have any effect on the exploration behavior and on the spatial knowledge of children or adults. In our second study (Jansen-Osmann et al., 2007b) we investigated the influence of the symmetry of the environment in more detail by using more directions and detour measurements between the start position and three landmarks. We provided additional evidence that the symmetry of the environmental structure indeed did not influence the acquired spatial knowledge as measured by direction estimations and distances walked in route knowledge and in detour tasks.

The results of the three studies showed that the environmental structure affected children only at an early stage of spatial knowledge acquisition, so that spatial knowledge may become increasingly independent of environmental structure over time. However, one piece of evidence is still missing. In our former two studies we varied the environment’s symmetry and its influence on all processing stages, i.e., exploration, learning, and spatial knowledge acquisition. In contrast, Herman et al. (1987) investigated only spatial knowledge acquisition in symmetrical environments with a square and curved structure. The present study was conceived to bridge the gap between our work and that of Herman et al. (1987) , i.e., to investigate the different processing stages in symmetrical environments with varying kinds of angles and paths. To get two symmetrical environments which can be compared in length and overall structure, a square environment and a circular one were constructed. We chose a virtual environment situation, which can be explored in a self-determined way (for a comprehensive discussion of the advantages and drawbacks of desktop virtual environments in spatial cognition research with children, see Jansen-Osmann & Fuchs, 2006; Jansen-Osmann & Wiedenbauer, 2004a; 2004b; 2004c). Although this has the disadvantage that the exposure to the environment cannot be strictly controlled, this method is closer to reality. The conducting of a developmental study is important because studies showed a developmental improvement at this age (for example Cohen & Schuepfer, 1980), a result which is confirmed by our own research (see e.g., Jansen-Osmann & Fuchs, 2006; Jansen-Osmann & Wiedenbauer, 2004a, 2004b, 2004c). 

## Method

### Participants

Eighty children from two age groups (7-8 and 11-12 years) and 40 adults participated in the study. The mean age of second graders was 7.62 years, that of the sixth graders 11.36 years, and that of the adults, who were students of the University of Düsseldorf, was 24.95 years. There were 20 females and 20 males in each age group. Children were recruited through advertisements in local newspapers asking for participation in a virtual environment experiment receiving a gratuity of 10 Euro. Prior to testing, all parents gave their informed written consent for participation in the study. The local ethics committee approved the experimental procedure.

### Materials

The study was conducted in a virtual world using the software 3D Game Studio. There were two symmetrical versions of the virtual world with either curved or straight routes (circular vs. square world). Both virtual mazes (see Figure 1) consisted of three main route-networks linked by eight routes which branched off at an angle of either 90° or 45°. As a consequence, at decision points routes branched off at an angle of either 0 (straight ahead), 90, 45 or 135° (see Jansen-Osmann, Schmid, & Heil, 2007a, Jansen-Osmann, Schmid, & Heil, 2007b). Because the shape of the surrounding area was not perceivable from the participant’s point of view, the construction of both virtual worlds was not confounded with the external frame of reference.

**Figure 1a.**
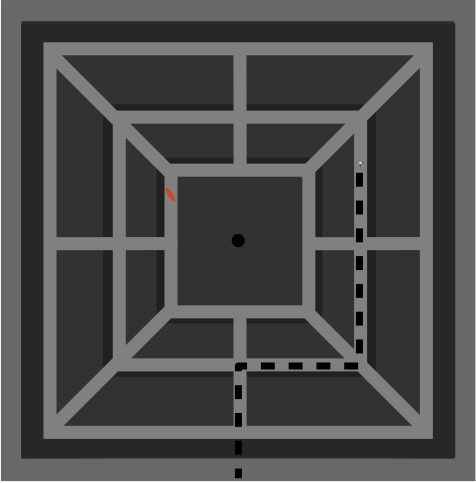
Figure 1a shows an overview of the square maze. The shortest route to reach the goal figuresismarked.

**Figure 1b.**
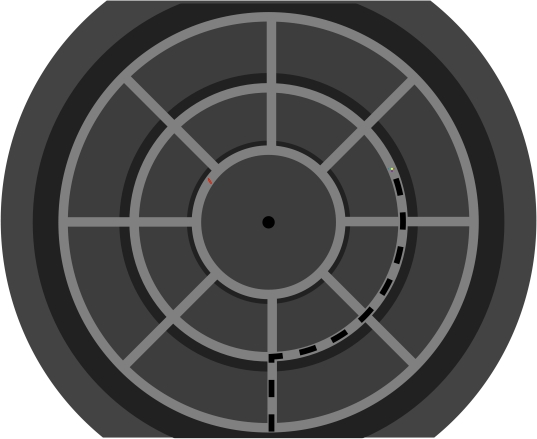
Figure 1b shows an overview of the circular maze. Only the maze’s interior was visible for the participants, i.e., they were not able to look over the outside walls.

The virtual world was presented from the first-person perspective and was projected onto a 17-in. flat-screen monitor. The distance between the monitor and the participant was 0.5 m. Participants explored the simulated maze using a joystick. The start position was set in a small cul-de-sac with brown walls.

### Procedure

Individual test sessions lasted about 30 min and took place in a laboratory at the Heinrich-Heine-University of Düsseldorf. Firstly, all participants were given the opportunity to practice handling the joystick by navigating through another (non experimental) virtual environment. This familiarization phase took approximately 5 min for each participant. Virtual walking speed approximated real-life walking speed. The joystick had to be pushed until dead-stop so that velocity was constant. Rotation and translation velocities were the same. Participants from each age group were randomly assigned to one of the virtual mazes (square vs. circular). There were three experimental phases (exploration, learning and spatial knowledge acquisition, or test phase). In the exploration phase, subjects were familiarized with the maze. The learning phase was assumed to shed light on the spatial learning, while the measurements of the test phase assessed the subject’s spatial knowledge. During all experimental phases, each participant’s position was recorded six times per second while they moved through the virtual maze, and their paths taken in each trial were plotted onto an overview (e.g., see Figure 1 in which the route walked by one participant in the exploration phase is marked). This allowed registering the distance walked in units of the software and retracing the route walked.

#### Exploration phase

Participants received the following instruction:

Now you have to explore an unknown virtual environment with two objects which you have to find: Bob the Builder and a fish. Please push the joystick until dead-stop and try to explore the whole maze. This phase will end after 5 minutes. If you do not find both objects within this time you can go on until you find them. If you find the objects earlier, please continue to explore the maze for the period of 5 minutes.

Because participants navigated in a self-determined way, the exact path used during the exploration phase varied between participants. The behavior in the exploration phase was measured by the distance walked.

#### Learning phase

After the exploration phase participants received the following instruction:

You have to explore the maze again, but now it is your task to find both goal toy-figures, namely Bob the Builder and the fish once again. You have to find the shortest route from the start-position to both target figures in two consecutive trials.

This shortest route (see Figure 1 for the goal object “Bob”) was defined as the one with the least distance to be walked and which consisted of two turns only in the square world and one turn in the circular world. Only one correct route for each object was possible: Participants had to turn right at the second intersection and left at the next intersection to reach the first goal object “Bob”. They had to turn left at the second intersection and right at the next intersection to reach the second goal object “fish”. All other possible routes were longer or had more turns. In contrast to the exploration phase, in which the task was merely to explore the maze, the spatial learning behavior in the learning phase was constrained, that is, the target figures had to be reached by choosing one or two turns only, which was defined as a learning criterion. Previous studies had shown that this learning criterion was very easy to understand even for the younger children (Jansen-Osmann & Wiedenbauer, 2004b).

The learning behavior was measured by the number of trials needed to achieve the learning criterion in the learning phase. Each walk from the start position until the target figure was reached was defined as one trial.

#### Spatial knowledge acquisition phase

After achieving the learning criterion, the participants completed (1) the direction estimation task and (2) the detour task. First, the viewpoint of the participant was set at the start-position. Participants were then instructed to estimate the direction from the start position to the location of the goal object “Bob” by moving the joystick in the specific direction and then pressing the joystick button. Corrective rotations were allowed before pressing the button. The dependent variable was the angular difference between the estimated and the correct angle (direction estimation task). After pressing the joystick button a barrier appeared which blocked the originally shortest route. Participants had to find a detour (i.e., an alternative short route) from the start position to the goal object “Bob”. When they arrived at this goal object they had to estimate the direction to the start (see above). Again, a barrier blocked the shortest route to the start and the participants had to find the shortest detour. The whole procedure was replicated with the “fish” as the goal object.

#### Test phase

The following four variables were analyzed in the test phase:

 1. Mean absolute error of the direction estimation from the start-point to the two goal objects. 

 2. Mean absolute error of the direction estimation from the two goal objects to the start-point. 

 3. Mean difference between the shortest path from the start-point to the two goal objects and the distance actually walked. 

 4. Mean difference between the shortest path from the two goal objects to the start-point and the distance actually walked. 

Because the optimal distance of the shortest paths from the start to the two goal objects differed slightly between the circular and the square maze, the shortest path was determined separately for each experimental condition. The correct direction estimation from the two objects to the start position and vice versa did not differ between the two environments.

In the test phase, participants were asked to fulfill the walking task between the two objects. They had to find the shortest route from one goal object (Bob) to the other (fish). For that, their viewpoint was set in front of the former.

To analyze the performance in the four detour tasks and the survey knowledge task, the distance walked was registered in units of the software (SU). After that, the walked distance was subtracted from the optimal distance in the two different virtual worlds. The experimental factor direction – start to goal object “Bob” (Detour 1), goal object “Bob” to start (Detour 2), start to goal object “fish” (Detour 3), and goal object “fish” to start (Detour 4) – was introduced for the analysis of the detour task. The other three factors in the learning and test phase were age group (younger children, older children and adults), type of maze (square, circular), and object (Bob, fish). Bonferroni follow-up tests were used in the statistical analysis. Half of each age group took part in each virtual environment.

Although gender differences have sometimes been found in spatial cognition research (Devlin & Bernstein, 1995; Lawton, 1994), our own research revealed a completely undifferentiated picture regarding spatial performance and knowledge in a virtual environment. On the one hand no gender differences were obtained at all (Jansen-Osmann & Wiedenbauer, 2004b), while on the other hand gender differences favoring men were found during map-tasks. In these tasks participants have to draw either the position of the goal object within the map, a map of the environment (Jansen-Osmann & Fuchs, 2006; Jansen-Osmann et al., 2007a), or the position of landmarks within the map (Jansen-Osmann & Wiedenbauer, 2004a). Because both measurements were not relevant for this study, gender was not regarded as an experimental factor. Furthermore, computer experience was not further analyzed because all of our other studies did not show any influence of computer experience on the measurements obtained (compare Jansen-Osmann & Fuchs, 2006; Jansen-Osmann et al, 2007a, 2007b; Jansen-Osmann & Wiedenbauer, 2004a, 2004b, 2004c).

## Results

The statistical significance level was set at α = .05.

### Exploration phase

The distance walked in the exploration phase was analyzed to make sure that differences in spatial knowledge were not attributable to differences in exploration behavior. There was no significant difference in the distance walked between age groups, *F*(2,108) = 0.2, η^2^ = .005, and type of maze, *F*(1, 108) = 0.4, η^2^=.004. Moreover, there was no significant interaction between age group and type of maze, *F*(2, 108) = 0.1, η^2^=.002.

### Learning phase

#### Number of learning trials

The analysis of variance revealed only a main effect of type of maze, *F*(1, 108) = 18.9, *p* < .001, η^2^ = .143. Only a marginally significant main effect of age group, *F*(2, 108) = 2.8, *p* = .065, η^2^ = .047 was found. There was no statistical main effect of object, *F*(1, 108) = 0.9, η^2^ = .00. Furthermore, there was neither a significant interaction between type of maze and age, *F*(2, 108) = 1.2, η^2^= .022; type of maze and object, *F*(2, 108) = 1.4, η^2^ = .005; nor age group and object, *F*(2, 108) = 0.9, η^2^ = .00. The three-way interaction between all experimental factors was also not significant, *F*(2, 108) = 0.1, η^2^ = .004. In the square maze, participants (*m* = 2.08, *SE* = 0.17) needed more learning trials than in the circular maze (*m*=1.25, *SE* = 0.12). As a trend, younger children needed more learning trials (*m*=1.90, *SE* = 0.16) than older children (*m*=1.72, *SE* = 1.35) and adults (*m*=1.37, *SE* = 0.97).

### Spatial knowledge acquisition phase

#### Direction estimation

As in our former study (Jansen-Osmann et al, 2007b), it was much easier for all participants to estimate the direction from the start to one of the two goal objects than vice versa – “Bob”, *F*(2, 108) = 8.4, *p* < .01., η^2^ = .073; and “Fish”, *F*(2, 108) = 15.2, *p* < .001., η^2^ = .125. Because no interactions between the factor direction of estimation and the other experimental factors were found, data presented were averaged across both directions.

The analysis of variance revealed a main effect of age group, *F*(2, 108) = 9.8, *p* < .001, η^2^ = .149. Neither statistically significant main effects of type of maze, *F*(1, 108) = 0.7, η^2^ = .006, and object, *F*(1, 108) = 0.7, η^2^ = .001, were found, nor significant interactions between type of maze and object, *F*(2, 108)= 0.4, η^2^ = .007; age group and object, *F*(2, 108) = 0.2, η^2^ = .025; and age group and type of maze, *F*(2, 108) = 2.8, η^2^ = .048. Similarly, the three-way interaction between all experimental factors was not significant, *F*(2, 108) = 0.3, η^2^ = .02. The absolute angle of direction estimation error was higher for the younger children (*m*=65.66, *SE* = 7.07) than for the older children (*m* = 41.94, *SE* = 4.29), which in turn was higher than that of the adults (*m* = 33.83, *SE* = 4.46).

#### Detour task

As in the direction estimation task we collapsed the data from the two detour tasks (start to goal object and vice versa). There was no difference in the two distance measurements – from start to the goal object and vice versa – for the route to goal object “Bob”, *F*(2, 108) = 2.6, η^2^ = .024, and the one to goal object “fish”, *F*(2, 108) = 0.0, η^2^ = .000.

The analysis of variance revealed main effects of object, *F*(1, 108) = 5.4, p < .05, η^2^ = .046, type of maze, *F*(1, 108) = 12.6, *p* = .001, η^2^ = .101, and age group, *F*(2, 108) = 8.1, *p* = .001, η^2^ = .126. No statistically significant influence was found for interactions between type of maze and age group, *F*(2, 108) = 0.3, η^2^ = .735; object and type of maze, *F*(2, 108) = 0.2, η^2^ = .012; object and age group, *F*(2, 108) = 0.8, η^2^ = .003; and the three-way interaction between all experimental factors, *F*(2, 108) = 0.4, η^2^ = .019. The distance walked was higher for the route to the goal object “fish” (*m*=2784.56, *SE* = 226.93) than for the route to the goal object “Bob” (*m*=2207.67, *SE* = 204.47), see Figure 2a. Moreover, it was higher for the younger (*m*=3388.73, *SE* = 341.33) than for the older children (m = 2295.26, *SE* = 283.34), which was higher than that of the adults (m = 1821.59, *SE* = 146.68), see Figure 2b. Furthermore, all participants walked substantially smaller detours in the circular maze (*m*=1912.20, *SE* = 209.12) than in the square one (m = 3079.99, *SE* = 268.95), see Figure 2c.

**Figure 2a.**
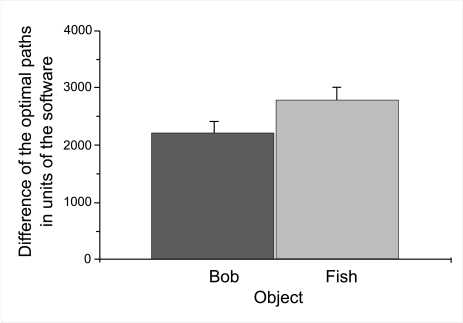
Mean deviation of distance walked from the optimal path dependent upon kind of object. Error bars indicate standard errors.

**Figure 2b.**
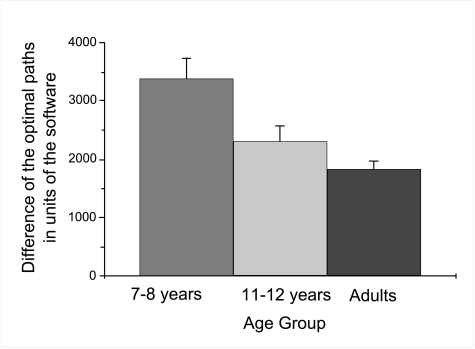
Mean deviation of distance walked from the optimal path, dependent upon age group (Figure 2b). Error bars indicate standard errors.

**Figure 2c.**
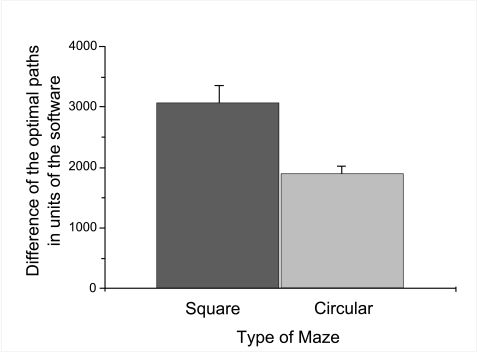
Mean deviation of distance walked from the optimal path, dependent upon type of maze. Error bars indicate standard errors.

#### Walking task between the two objects

Concerning the distance walked between the two objects in the maze, a univariate analysis of variance revealed a significant influence of the factors type of maze, *F*(1, 108) = 11.7, *p* = .001, η^2^ = .095, and age group, *F*(2, 108) = 4.5, *p* < .05, η^2^ = .075. No significant interaction between type of maze and age group was found, *F*(2, 108) = 0.7, η^2^ = .012. Participants walked longer distances in the square maze (*m*=2569.13, *SE* = 280.55) than in the circular maze (m = 1317.23, *SE* = 246.85), see Figure 3a, and younger children (*m*=2574.05, *SE* = 399.55) walked longer distances than older ones (*m*=2033.30, *SE* = 350.60) and adults (*m*=1240.22, *SE* = 211.42), see Figure 3b.

**Figure 3a.**
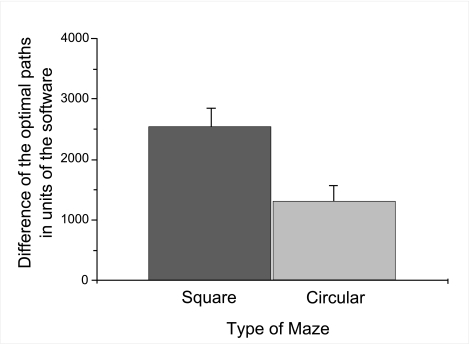
Mean deviation of distance walked between Bob and Fish dependent upon type of maze. Error bars indicate standard errors.

**Figure 3b.**
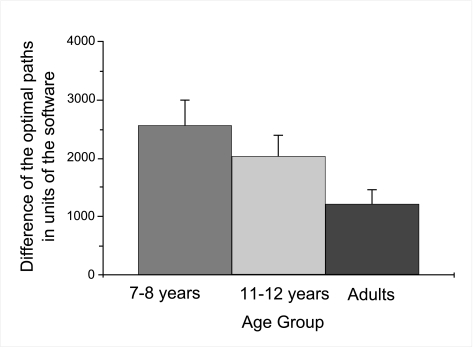
Mean deviation of distance walked between Bob and Fish dependent upon age group. Error bars indicate standard errors.

## Discussion

Our results provide a clear picture: The learning of a route was superior in a circular world than in a square world, meaning that all participants needed fewer learning trials to achieve the criterion in an environment with curved routes. Furthermore, only in the circular world did participants of all age groups walk shorter distances from the start to the goal objects and vice versa, and on the route between the two landmarks inherent in the maze. There was no such advantage of the circular world concerning the exploration behavior and the task to estimate the direction between the goal objects and the start position. Furthermore, a developmental achievement from childhood to adulthood was found in all measurements of the spatial knowledge acquisition phase, but not in the exploration and learning phase.

Given these results we can conclude that the environmental structure indeed influenced different processing stages in the spatial knowledge acquisition for both school-aged children and adults. At first glance this seems to be in contrast with the results of our former studies where no such influence was found (Jansen-Osmann et al, 2007b) or where the influence was restricted to the learning phase of the youngest children (Jansen-Osmann et al., 2007a). But compared to these former studies, it was not the symmetry of the environmental structure that was varied in the present investigation, but the kind of angles and paths: Whereas the square environment was built by right angles and the combination of 45° or 135° angles, there were no such angles in the circular environment. Thus the results of the present study extend those of Herman et al. (1987) . This is because investigating different processing stages in the spatial cognition process and allowing a free exploration of the environment indeed revealed the influence of the environmental structure on three of the four different measures. The advantage of the circular world was not present for the direction estimation task but only for the task to find a route and the distance walked. One might assume that in the learning phase and the distance walked task information was tied more strongly to one’s own body- or viewpoint position than in the task to estimate the direction of objects. For that, spatial learning and distance walked tasks differed in some way from the direction estimation tasks. 

But why is it easier to acquire spatial knowledge in a circular environment than in a square one? One may speculate that the concept of angles is quite arbitrary and does not really help us to orientate ourselves, even though it does not interfere, when we explore an environment for the first time (exploration behavior). But this assumption is in contrast to the observation that people like to straighten curved paths in memory (compare Montello, 1991). Certainly, that is narrative, but just recently it was shown that humans prefer curved visual objects in comparison to objects which are angled (Bar & Neta, 2006). Furthermore, we know from environmental psychology that the criteria of the “fewest turns” is one of the most often used criteria in route selection (Golledge, 1995). This might give a hint that people like to choose routes with fewer turns, and that turns are not as prominent as perhaps previously assumed. When people were asked what criteria they usually chose when selecting routes in their real world activity, criteria such as “most aesthetic” and “many curves” were not mentioned as often as they were used in an experimental route selection task (Golledge, 1995).

Additionally, differences due to age increased with the processing stage in the spatial cognition process: There was no age effect in the exploration behavior and only a marginally significant effect in the learning behavior. A developmental achievement from childhood to adulthood was only observed in all measurements of the test phase, the spatial knowledge measurements. The aim of the present study was to evaluate how spatial knowledge develops out of the behavior in a new environment, in this case a virtual one. The results give a first hint that differences in behavior in an unknown environment might not be caused by age effects. Instead, the cognitive processes themselves may differ between children and adults. This is in accordance with a study of Allen and Ondracek (1995) , where the relationship between age-sensitive cognitive abilities and children’s acquisition of spatial knowledge was emphasized (e.g., perceptual-motor speed mediated the relationships between age and route knowledge). 

At present, it is difficult to decide whether the age differences in the test phase were due to general cognitive development or due to spatial cognitive development only. As children become older their ability to divide space into smaller categories improves, which helps them to act in the environment and to represent spatial information. One might speculate that in an environment with only a little landmark information the environmental structure plays the main role, and hierarchical coding processes might dominate resulting in the age differences obtained here.

Finally, the robustness of the findings and the generalization using the desktop system has to be discussed. Studies are needed which directly compare knowledge acquisition in real and virtual environments under a developmental perspective. There are adult studies investigating – both in real and virtual environments – the most important properties of the spatial representations underlying spatial behavior (Loomis, Blascovich, & Beall, 1999). In these studies, both environments led to similar results (Péruch & Wilson, 2004; Tlauka, 2007). However, there is also evidence questioning the ecological validity of desktop virtual environments (Hegarty, Montello, Richardson, Ishikawa, & Lovelace, 2006). With the exception of three studies (Laurance, Learmonth, Nadel, & Jacobs, 2003; Plumert, Kearney, & Cremer, 2004), this comparison, however, is still missing in studies with children. Interestingly, Laurance et al. (2003) showed that children used the virtual space as if it was real. Comparing the different processing stages in virtual and real space with a group of 120 participants (40 children at the age of 7-8, 40 children at the age of 11-12, and 40 adults), we obtained evidence that spatial behavior and knowledge acquisition indeed is comparable in both environments. 

## Conclusion

The present study investigated the influence of the environmental structure on spatial knowledge acquisition in a large-scale space in children and adults. The main result was that the degree to which a route was straight or curved influenced spatial learning for participants of each age group. We obtained age differences in all spatial tasks but not for exploratory behavior. This might indicate that cognitive development in general, and not spatial cognition in particular, is important for spatial learning in a large-scale environment.

Even though the results reported here are quite promising, some questions should be addressed in more detail. These concern the influence of different variations of the environmental structure (i.e., symmetry, regularity, and type of angles) on spatial knowledge acquisition and the importance of circular concepts in spatial cognition.
